# Consistent individual differences in seed disperser quality in a seed-eating fish

**DOI:** 10.1007/s00442-016-3749-4

**Published:** 2016-10-04

**Authors:** Bart J. A. Pollux

**Affiliations:** Experimental Zoology Group, Department of Animal Sciences, Wageningen University, De Elst 1, 6708 WD Wageningen, The Netherlands

**Keywords:** Ichthyochory, Intra-specific variation, Long-distance dispersal, Seed retention time, Zoochory

## Abstract

Animal-mediated seed dispersal (zoochory) is considered to be an important mechanism regulating biological processes at larger spatial scales. To date, intra-specific variation in seed disperser quality within seed-dispersing animals has not been studied. Here, I employed seed feeding trials to quantify individual differences in disperser quality within the common carp (*Cyprinus carpio*) using seeds of two aquatic plants: unbranched bur-reed (*Sparganium emersum*, Sparganiaceae) and arrowhead (*Sagittaria sagittifolia*, Alismataceae). I found substantial variation among carp individuals in their propensity to ingest seeds and their ability to digest them, resulting in up to 31-fold differences in the probability of seed dispersal. In addition, there were significant differences in the time that seeds are retained in their digestive systems, generating a twofold difference in the maximum distance over which they can potentially disperse seeds. I propose that seed-eating animal species consist of individuals that display continuous variation in disperser quality, with at one end of the continuum individuals that are likely to eat seeds, pass them unharmed through their digestive tract and transport them over large distances to new locations (i.e. high-quality seed dispersers) and at the other end individuals that rarely eat seeds, destroy most of the ones they ingest and transport the few surviving seeds over relatively short distances (low-quality seed dispersers). Although individual differences in seed dispersal quality could be the result of a variety of factors, these results underline the ecological and evolutionary potential of such variation for both plants and animals.

**Electronic supplementary material:**

The online version of this article (doi:10.1007/s00442-016-3749-4) contains supplementary material, which is available to authorized users.

## Introduction

Natural populations often consist of individuals that exhibit significant phenotypic variability in morphological, physiological or behavioural attributes (Bolnick et al. 2003; Careau et al. 2008; Bergmüller and Taborsky 2010; Biro and Stamps 2010; Araújo et al. 2011; Violle et al. 2012). Such intra-specific variation was long considered to be a troublesome source of variation in ecological studies; however, recent insights highlight the ecological and evolutionary significance of individual specialization within populations (Bolnick et al. 2011; Burton et al. 2011; Edelaar and Bolnick 2012; Edelaar et al. 2012; Wolf and Weissing 2012).

Seed dispersal is an example of a phenotypic trait that varies within plant species. The seeds of a single plant are dispersed over a range of distances away from the parent. This range is modelled by dispersal kernels, which typically have a positively skewed leptokurtic shape: Most seeds end up in close proximity to the parent while a smaller proportion, represented by the extended tail of the distribution, is dispersed over greater distances (i.e. long-distance dispersal; Nathan and Muller-Landau 2000). Although relatively rare, long-distance dispersal is essential for biological processes that take place on larger spatial scales, including metapopulation dynamics, resilience of regional communities or biological invasions (Cain et al. 2000; Nathan et al. 2003; Nathan 2006). Animal-mediated seed dispersal (zoochory) is considered to be a particularly important mechanism for long-distance dispersal, because animals can move fast, in a directional manner and are likely to transport seeds across dispersal barriers (Wenny and Levey 1998; Purves and Dushoff 2005; Purves et al. 2007; Nathan et al. 2008; D’hondt et al. 2012; Bauer and Hoye 2014).

**Fig. 1:**
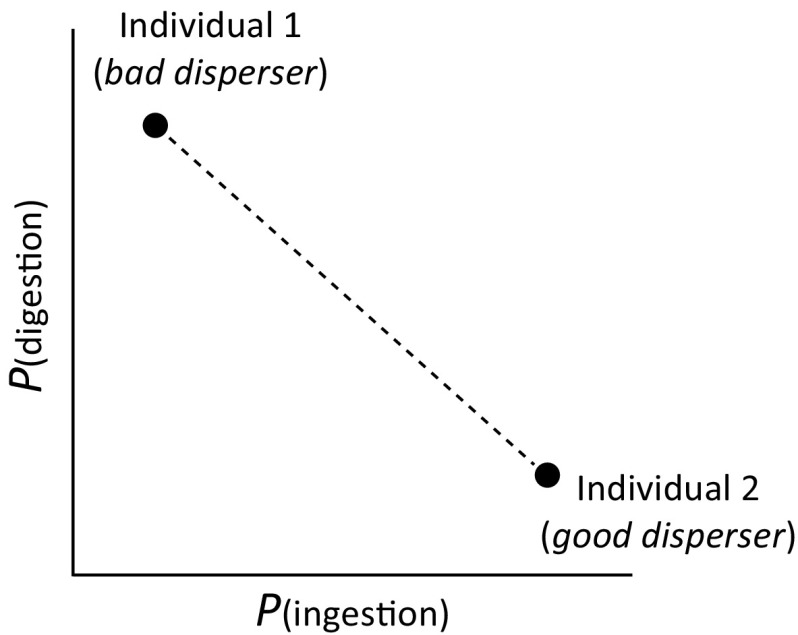
Intra-specific variation in disperser quality in a single population comprising two hypothetical individuals at opposing ends of a disperser quality continuum. The two individuals are represented by *separate dots*. Individual 1 has a low disperser quality, characterized by a low probability of seed ingestion and a high probability of seed digestion translating into a low overall likelihood of seed dispersal. Individual 2 has a high disperser quality, typified by a high probability of seed ingestion and low probability of digestion resulting in a high overall likelihood of seed dispersal

Successful seed dispersal via animals is the culmination of intimate plant–animal interactions. Whilst it has long been known that large inter-specific differences in the ability to disperse seeds exist among animal species (e.g. Howe and Smallwood 1982; Howe 1986; Schupp 1993), to date it remains unclear if, and to what extent, intra-specific variation in disperser quality exists among individuals within a single species. Animals may vary intra-specifically in their propensity to ingest the seeds they encounter in the field (Wilson et al. 1990) or their ability to digest them. Such individual differences affect the likelihood that seeds are transported intact to new locations. Figure 1 illustrates a simple case of individual variation in disperser quality among animals with two hypothetical individuals at opposing ends of a disperser quality continuum: Individual 1 is representative of a ‘bad disperser’, having a low probability of ingesting seeds when encountering them in the field and a high probability of digesting them. Individual 2, on the other hand, typifies a ‘good disperser’, having a high probability of ingestion and a low probability of seed digestion. Individuals may further vary in the duration that seeds are retained in their guts (Vander Noot et al. 1967; Sun et al. 1997), affecting the potential distance over which seeds can be dispersed. In general, the longer seeds remain in the digestive tract the greater the potential dispersal distance (Pollux 2011), although the actual shape and scale of a dispersal kernel is ultimately determined by the movements of the dispersers (Vellend et al. 2003; Westcott et al. 2005; Russo et al. 2006). Many captive experiments with fishes, birds and mammals show that, in single feeding trials, there is much individual variation in seed ingestion, retention time and gut survival between individuals (reviewed in Online Resource 1). However, none of these studies repeated the experiments and, hence, it remains unclear whether the observed variation is consistent (i.e. repeatable over time).

In this study I employ repeated seed feeding trials to study the extent and consistency of intra-specific variation in disperser quality within the common carp (*Cyprinus carpio*) using seeds of two aquatic plant species: unbranched bur-reed (*Sparganium emersum*, Sparganiaceae) and arrowhead (*Sagittaria sagittifolia*, Alismataceae). Recent studies highlight the prevalence of seed dispersal by fishes (ichthyochory) (Correa et al. 2007; Horn et al. 2011; Correa et al. 2015b). Worldwide, over 275 fish species have been shown to consume seeds in the field and, in the neotropics alone, at least 566 plants species (from 82 families) have been identified whose seeds constitute part of a fish’s diet (Horn et al. 2011; Correa et al. 2015b). The ecological relevance of ichthyochory for high-quality, long-distance seed dispersal (Anderson et al. 2009, 2011; Van Leeuwen et al. 2016), the maintenance of genetic diversity (Markwith et al. 2009; Pollux et al. 2009a) and the structuring of vegetation along rivers (Gottsberger 1978; De Souza-Stevaux et al. 1994; Horn 1997; Horn et al. 2011; Correa et al. 2015a) is also rapidly gaining recognition.

In the feeding trials employed in the present study, the endo-zoochoric dispersal process is divided in a number of discrete, consecutive steps that can be individually quantified (Charalambidou and Santamaría 2002; Pollux 2011), namely: (i) seed ingestion, the probability that offered seeds are ingested, which is affected by both seed availability and the animals’ feeding preferences; (ii) retention time, the time that seeds are retained in the digestive system of the animal; (iii) seed survival, the probability that seeds pass intact through the intestinal tract of animal dispersers and (iv) seed viability, the probability that recovered seeds are able to germinate. There is currently little or no empirical information about the existence of individual differences in disperser quality within seed-eating animal species. Here, I test whether: (1) animals vary intra-specifically in their ability to disperse seeds; (2) whether this variation occurs only in one, or in multiple stages, of the dispersal process (i.e. ingestion, retention time, survival, germination) and (3) if this individual variation is repeatable (consistent) over time, using the common carp as a model species.

## Materials and methods

### Study species

*Cypriniform* fishes represent the dominant seed-eating fish lineage in the Palearctic (i.e. Europe, northern Asia, northern Africa and the northern and central parts of the Arabian Peninsula) and Indomalayan (e.g. India, southern Asia, Indonesia, the Philippines, Taiwan) ecozones (Horn et al. 2011). The common carp’s native range extends to the rivers draining into the Black, Caspian and Aral Sea (Koehn 2004). To date it is the most widely spread cyprinid fish species in the world being commonly found in lakes, canals and lowland rivers in temperate and tropical regions of Eurasia, Africa, Australia and North America. Dietary studies show that the common carp is an opportunistic omnivore that forages on a variety of food items (e.g. seeds, fruits, plants and plant-associated invertebrates; Ridley 1930; Crivelli 1981; Bergers 1991; Garcıa-Berthou 2001; Horn et al. 2011; Grutters et al. 2015). Twelve common carps with a mean (±SE) mass of 0.307 ± 0.01 kg (range 0.260–0.359 kg) were obtained from Ruud Vonk Fish Hatchery (Maurik, The Netherlands) in October 2003 (Pollux et al. 2006). The fish were individually kept in 100-L tanks in a common garden setting at the fish facilities of Radboud University Nijmegen, The Netherlands. The water in the tanks was maintained at 24 °C and was continuously aerated (with airstones) and refreshed (50 l h^−1^). To ensure homogenization of water quality among the 12 tanks, all tanks were supplied with water coming from the same filtering system. The fish were kept on a diet of commercial Trouvit pellets (with a pellet diameter of 2 mm) up to a daily amount of 1 % of their body mass (Trouvit, Trouw & Co, Putten, The Netherlands).

Unbranched bur-reed (*S. emersum*) and arrowhead (*S. sagittifolia*) are aquatic, facultatively clonal, vascular macrophytes that are widely distributed throughout Eurasia and North America (Cook and Nicholls 1986; Pollux et al. 2007a) where their distribution overlaps with that of the common carp. They typically grow in a wide band at the margins of canals, rivers and streams that are characterized by shallow, slow-flowing waters. Both species flower from June to August (Sargent and Otto 2004). Their fruits (hereafter called seeds) are released in autumn. They remain dormant during the winter during which they are dispersed by water currents (Boedeltje et al. 2004; Pollux et al. 2009b), fish (Ridley 1930; Pollux et al. 2007b; Boedeltje et al. 2015) and waterfowl (Pollux et al. 2005; Soons et al. 2016), until they germinate in the following spring. Ripe seeds were collected during October 2003 from natural populations in The Netherlands (Pollux et al. 2006). The seeds were stored in glass jars filled with tap water, in a dark cold room at 5 ± 1 °C at the Radboud University Nijmegen (The Netherlands), to mimic the natural cold-stratified conditions of Central-North European winters required to break seed dormancy (Muenscher 1936).

### Experimental design

The common carp is an opportunistic omnivore that predominantly takes up seeds while foraging on vegetative plant parts, or while sifting through detritus in search for invertebrate prey (Horn et al. 2011; Pollux 2011). In the present study the process of ‘accidental’ uptake was mimicked by hiding the seeds in food pellets (cf. Horn 1997). Three weeks prior to the start of the first feeding trial, the carp were trained to voluntarily ingest these self-made food pellets, each containing five *S. emersum* and five *S. sagittifolia* seeds. The food pellets were prepared by adding warm water to commercial Trouvit pellets until the mixture procured a ‘doughy’ consistency. Five randomly selected *S. emersum* and five *S. sagittifolia* seeds were then added to a small amount of ‘Trouvit dough’ and kneaded into small pill-shaped balls with a diameter of approximately 10 mm (see Online Resource 2a). The pellets were subsequently placed in a drying stove and left to dry and harden for 24 h at a temperature of 28 °C.

To test for individual differences in seed disperser quality among the carp, I employed seed feeding trials. With each of the 12 fish individuals I performed 12 repeated feeding trials at weekly intervals (during January to April 2004), yielding a total of 12 × 12 = 144 separate trials. All trials started at 8:00 am in the morning, the time at which the carp were accustomed to receiving their daily food. At the beginning of each trial the self-made food pellets were offered to the carp, one pellet at a time (Online Resource 2b), until they were satisfied up to a maximum of ten food pellets (equivalent to a maximum of 50 *S. emersum* and 50 *S. sagittifolia* seeds). Five to ten minutes after feeding, non-ingested seeds (i.e. seeds that were expelled by ‘spitting’; Sibbing et al. 1986) were removed from the tanks with aquarium nets (gape size 10 × 15 cm; square mesh size 1 mm) and counted. Next, for a period of 24 h fish faeces were collected every 2 h from the bottom of the tanks by means of aquarium nets (preliminary tests, lasting 48 h, showed that the fish always excreted all non-digested seeds well within 24 h). Collected faeces were immediately rinsed with tap water and sieved using a 500 μm square mesh size sieve (diameter 19 cm) and retrieved seeds were counted. Retrieved seeds were transferred to plastic containers (100 mL) filled with tap water and returned to the dark cold room (5 ± 1 °C) for the remainder of the experiment to ensure an equal cold-stratification period for all seeds in all feeding trials (from seed collection in the field in October 2003 to the germination test in May 2004). In May 2004, all seeds were set to germinate simultaneously in a climate chamber with a photoperiod of 16L:8D (light:dark), a daytime irradiance of 200 μmol photons s^−1^ m^−2^ and a day/night temperature cycle of 25/18 °C. Seeds were placed in transparent polystyrene microtiterplates (127 × 82 cm, 96 wells; Omnilabo International BV, Breda, The Netherlands), filled with tap water (one seed per well). Germination, in our study defined as the emergence of the first foliage leaf, was checked daily for a period of 45 days (Pollux et al. 2006).

### Statistical analyses

The probability of seed ingestion (proportion of offered seeds that were ingested), survival (proportion of ingested seeds recovered from the faeces) and germination (proportion of recovered seeds that germinated by the end of the germination run) was assessed by fitting generalized linear models to the data for each plant species separately, using the GLIMMIX procedure in SAS 9.3 (SAS Institute Inc., Cary, NC, USA) with fish individual included as a fixed factor, feeding trial as a random factor and a binomial distribution and logit link function for the response variables. The GLIMMIX procedure uses the number of offered, ingested and survived seeds as the binomial denominator, thereby controlling for the effects of sample size in the analyses on the probability of ingestion, survival and germination, respectively (Littell et al. 2006). Due to the sequential nature of the three response variables in the study design the sample sizes, and therefore the degrees of freedom, tended to decline over the course of each feeding trial. Models were qualitatively evaluated based on the dispersion parameter, calculated by dividing the deviance by its degrees of freedom (deviance/*df*). When the dispersion parameter approaches 1, standard errors and confidence intervals are assumed to be unbiased and correlations among observations accounted for (Littell et al. 2006; Morel and Neerchal 2012). When the dispersion parameter was larger than 1, the model was assumed to be overdispersed. Failure to correct for overdispersion can lead to erroneous inferences due to inflated Type I error rates. Therefore, the model was rerun taking the overdispersion into account by adding a multiplicative overdispersion parameter to the variance function (Littell et al. 2006; Morel and Neerchal 2012). Adding an overdispersion parameter does not alter any of the parameter estimates, but only adjusts the variance–covariance matrix of the estimates by a constant factor (Littell et al. 2006; Morel and Neerchal 2012). Pair-wise post hoc comparisons of means were subsequently used to evaluate differences with a sequential Bonferroni corrected comparison-wise error rate (Holm 1979; Rice 1989).

The degree of relationship of the probability of ingestion, survival and germination, respectively, between the two plant species was assessed by computing Pearson product-moment correlation coefficients (*r*) with Fisher’s z transformation using arcsin square-root transformed proportions (Sokal and Rohlf 2001).

Differences in retention time were tested in a survival analysis by fitting a Cox proportional hazards regression model to the retrieval time data (the time between ingestion and defecation of seeds, in hours) for each individual seed that was recovered from the faeces, using the PHREG procedure in SAS 9.2. For each plant species we fitted separate models with fish individual included as a fixed factor. Pair-wise post hoc comparisons of means were used to evaluate the significance of differences among fish individuals with a sequential Bonferroni corrected comparison-wise error rate (Holm 1979; Rice 1989).

## Results

**Fig. 2:**
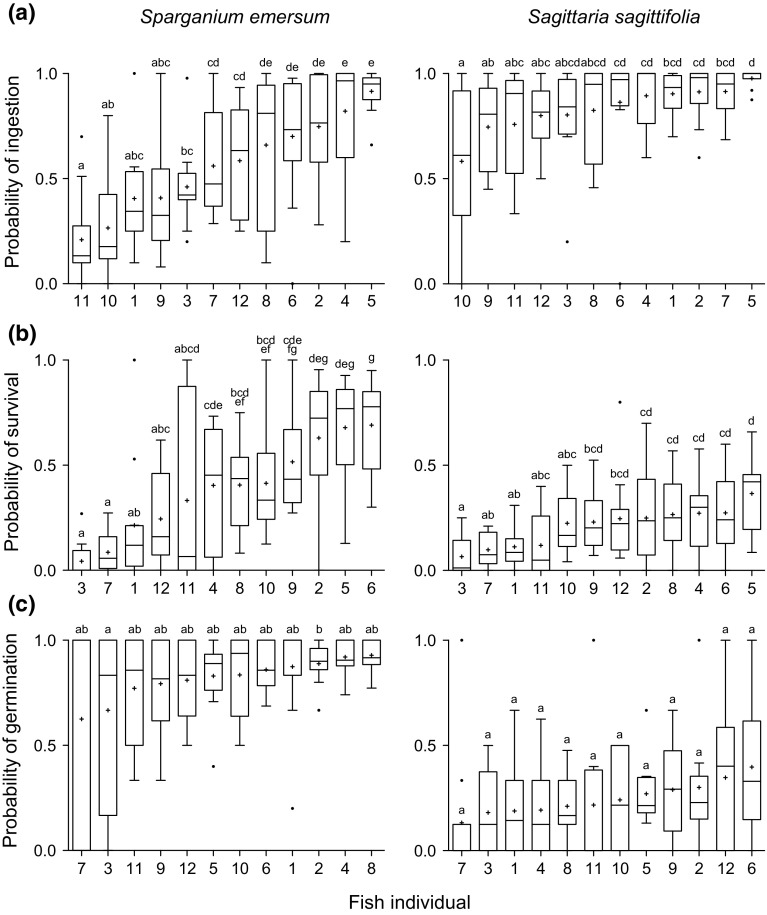
*Box* and *whisker plots* showing the probability of **a** ingestion, **b** survival and **c** germination of *Sparganium emersum* (*left panels*) and *Sagittaria sagittifolia* (*right panels*) seeds fed to 12 common carp (*Cyprinus carpio*). Each *box* and *whisker plot* represents the data of a single carp individual and is based on *N* = 12 repeated feeding trials. The *boxes* range from the 25th to the 75th percentile of the distribution, with the *horizontal line* in the *box* representing the median and the ‘*plus*’ sign signifying the mean. The *whiskers* extend to 1.5 times the interquartile range (IQR). Data points lying outside this range are represented by *black dots*. *Box* and *whisker plots* that do not share a common *letter* are significantly different from each other (post hoc comparisons with sequential Bonferroni corrected comparison-wise error rates). The *box* and *whisker plots* were created with Prism 5.0c (GraphPad Software Inc., La Jolla, CA, USA)

The probability of seed ingestion differed significantly among fish individuals, both for *S. emersum* (*F*_11,121_ = 13.78, *P* < 0.0001) ranging from a mean ± SE of 20.90 ± 6.10 % (individual 11) to 91.53 ± 2.95 % (individual 5; Fig. 2a) and *S. sagittifolia* (*F*_11,121_ = 6.03, *P* < 0.0001) where it ranged from 58.24 ± 10.51 % (individual 10) to 97.71 ± 2.10 % (individual 5; Fig. 2a), indicating that some individuals are consistently more inclined to consume seeds when offered to them than others. The positive correlation between the probability of ingestion of *S. emersum* and of *S. sagittifolia* (Pearson: *n* = 12, *r* = 0.79814, *P* = 0.0007) indicates that individuals that ingested more *S. emersum* seeds also ingested more *S. sagittifolia* seeds (Online Resource 3a).

The probability of seed survival also differed significantly among the 12 fish, both for *S. emersum* (*F*_11,117_ = 13.28, *P* < 0.0001) ranging from 4.33 ± 2.59 % (individual 3) to 69.05 ± 6.69 % (individual 6; Fig. 2b) and *S. sagittifolia* (*F*_11,118_ = 7.23, *P* < 0.0001) ranging from 6.60 ± 2.79 % (individual 3) to 36.59 ± 5.10 (individual 5; Fig. 2b), indicating that some individuals are far more efficient in digesting seeds than others. There was a positive correlation between the probability of survival of *S. emersum* and *S. sagittifolia* (Pearson: *n* = 12, *r* = 0.81889, *P* = 0.0004), suggesting that individuals that digested more *S. emersum* seeds also digested more *S. sagittifolia* seeds (Online Resource 3b).

**Fig. 3:**
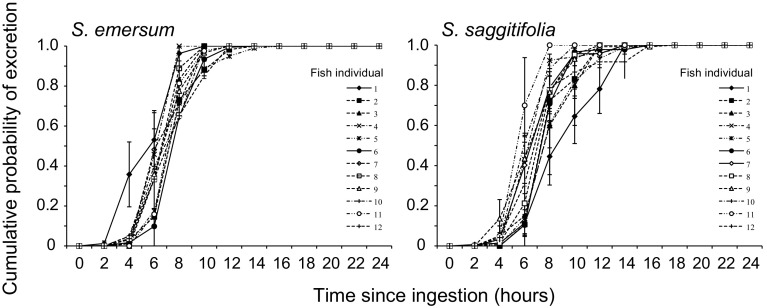
Mean (±SE) cumulative seed excretion over a period of 24 h (number of seeds retrieved after *t*_*n*_ hours after ingestion [*t*_0_]/total number of excreted seeds at *t*_24_) of *Sparganium emersum* and *Sagittaria sagittifolia* seeds fed to 12 common carp (*Cyprinus carpio*). The faeces were collected every 2 h. *Each line* represents a single carp individual and is based on *N* = 12 feeding trials. Post hoc comparisons between individuals were performed using sequential Bonferroni corrected comparison-wise error rates (given in Online Resource 4)

The pattern of seed retrieval over time followed a leptokurtic curve, with all seeds being retrieved within 16 h (Fig. 3). Cox proportional hazards regressions revealed significant differences in retrieval rate among fish individuals, both for *S. sagittifolia* (*χ* ^2^ = 42.338, *df* = 11, *P* < 0.0001) and *S. emersum* (*χ* ^2^ = 85.778, *df* = 11, *P* < 0.0001) (Fig. 3; for post hoc comparisons among individuals see Online Resource 4).

Although the probability of germination of *S. emersum* seeds did differ significantly among fish individuals (*F*_11,92_ = 2.84, *P* < 0.0030), ranging from 62.50 ± 16.14 % (individual 7) to 92.84 ± 2.10 % (individual 8; Fig. 1h), post hoc analyses revealed that only one individual (individual 3) was significantly different from one other individual (individual 2), while the remaining individuals did not significantly differ from one another; Fig. 2c). Moreover, the probability of germination of *S. sagittifolia* seeds did not differ significantly among any of the 12 fish (*F*_11,101_ = 1.72, *P* < 0.0795), ranging from 13.26 ± 9.21 % (individual 7) to 39.71 ± 9.83 % (individual 6; Fig. 2c). Finally, there was no correlation between the probability of germination of *S. emersum* and *S. sagittifolia* (Pearson: *n* = 12, *r* = 0.34496, *P* = 0.2593; Online Resource 3c). Together, these results suggest that differences in the digestive physiology among the carp individuals do not translate into clear differences in the germination probability of either plant species.

## Discussion

### Consistent individual differences

This study discloses remarkable variation among carp individuals in their propensity to ingest seeds of aquatic plants and their ability to subsequently digest them. This intra-specific variation is significant and repeatable over time (Fig. 2; Online Resource 5) and hence most likely reflects consistent individual differences (CIDs; Biro and Stamps 2010) in disperser quality among the carp. Such intra-specific variation may be caused by differences in genotype, phenotype, environment, social interactions or combinations of these factors (Santamaría et al. 2002; Sargeant 2007); however, the relative importance of ‘nature’ versus ‘nurture’ currently remains insufficiently understood. Disentangling the potentially confounding causes underlying the observed individual differences in seed disperser quality among carp will therefore require further investigation (e.g. parent–offspring regressions to estimate the heritability of seed disperser quality).

### Ingestion, survival and germination

Differences in seed ingestion between carp individuals results from seed selection in the fish’s oral cavity. Carp have highly complex intra-oral food selection mechanisms for the detection and investigation of potential food items. Purification of food is achieved by repetitive rinsing and resuspension in the oral cavity by orobuccal expansion, aided by pumping of water through the opercular valves. Edible items are separated from the remaining material through size-dependent selection of particles by the branchial sieve (with minimal mesh width ranging from 250 to 500 µm; Sibbing et al. 1986), followed by a quality-dependent selective “taste and sort” mechanism that separates palatable food from the remaining waste material using abundant taste buds lining the palatal organ (up to 820/mm^2^; Sibbing and Uribe 1985). Inedible particles are expelled by ‘spitting’, a reversed suction pump action of the orobuccal and opercular cavities, while palatable food items are retained and transported to the pharyngeal teeth and chewing pad in the posterior pharynx where they are subjected to mastication (crushing and grinding) and subsequently swallowed (deglutition) (Sibbing 1982; Sibbing and Uribe 1985; Sibbing et al. 1986; Sibbing 1988; Callan and Sanderson 2003). This study demonstrates that some individuals are more likely to qualify seeds as inedible food particles and expel them by ‘spitting’ than others. The underlying mechanism for this individual variation in seed selection requires further study, but is likely linked to differences in the structure of the branchial sieve (Sibbing et al. 1986) or the density of taste buds lining the palatal organ (Sibbing and Uribe 1985).

Seed survival also varied significantly among the carp, possibly due to differences in the morphology of the gut and/or intestinal mucosa (Dabrowski 1983; Lee and Cossins 1988) or the enzymatic activity of the digestive juices (Bondi and Spandorf 1954). Unfortunately, studies focussing on intra-specific variation in the gut morphology and enzymatic activity of the digestive juices are lacking in fishes. Differences in bite force exerted on the seeds during mastication (Sibbing 1988) among the carp are not likely to have played a role in the present study, because bite force is strongly correlated with fish size and differences in body weight among the experimental subjects (mean ± SE 307 ± 10 g, *N* = 12 carp) are insufficient to explain the observed variation in seed survival (Nand Sibbing, personal communication).

Fish individual had no effect on the germination of *S. sagittifolia* seeds and only a minor effect on *S. emersum* seeds. These two plant species both produce non-fleshy fruits: *S. emersum* produces a drupe-like fruit consisting of a seed enclosed in a hard scleridial endocarp and a tough spongy mesocarp, while *S. sagittifolia* produces a nutlet-like seed surrounded by a soft membranous endocarp and a semi-transparent, laterally compressed disc-like mesocarp (Pollux et al. 2006). In these species, any effect of gut passage on seed germination (either positively or negatively) is most likely caused by the mechanical or chemical treatment (scarification) of the seed coat in the animal’s gut (Traveset 1998; Traveset and Verdú 2002; Pollux et al. 2006; Traveset et al. 2007). This study therefore suggests that intra-specific differences in gut structure or digestive physiology of carp are not large enough to cause substantive differences in seed scarification. Whilst I did not measure the gut length of our carp, it is known that the length of their intestine correlates with body size: e.g. it has been shown to vary from 50 cm in carp that weigh 300 g up to 80 cm in carp of 700 g (Dabrowski 1983). Given the limited variation in body weight among the experimental subjects used in this study, however, an effect of gut length on germination was not expected.

### Individual differences in disperser quality

**Fig. 4:**
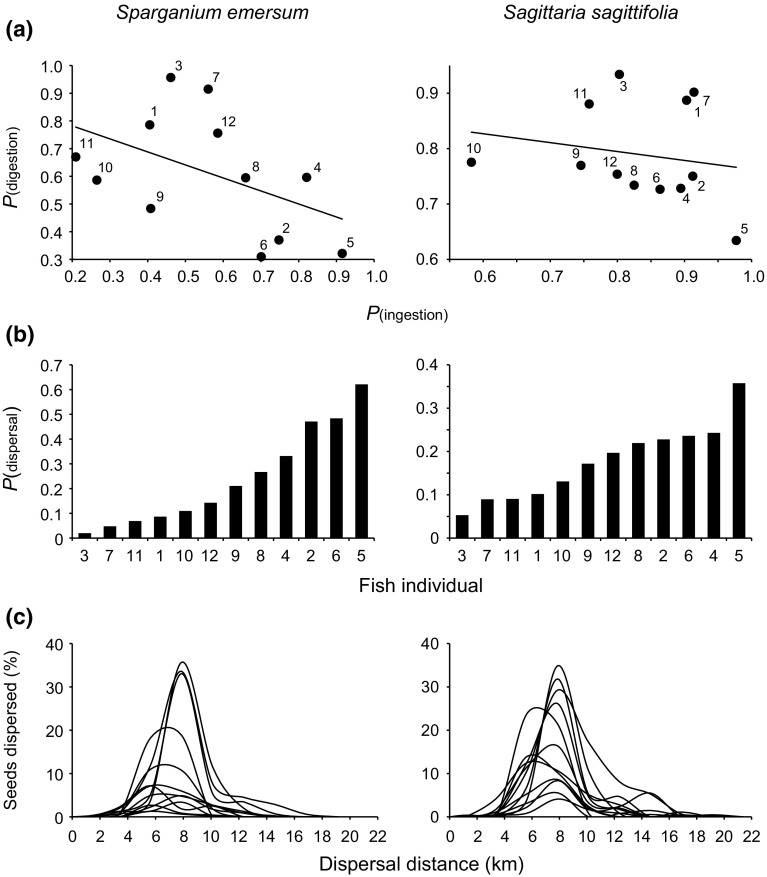
Summary of individual differences in disperser quality among the common carp (*Cyprinus carpio*). **a** Graphical representation of individual differences in fish-mediated dispersal of *Sparganium emersum* (*left*) and *Sagittaria sagittifolia* (*right*) seeds among 12 carp. The *dots* represent different carp individuals (the *numbers* next to the *dots* identify the exact individual). The position of the *dots* in the graph conveys information about individual disperser quality: Carp individuals at the *top left* corner of the graph rarely eat seeds and are likely to digest them (low *P*_[ingestion]_, high *P*_[digestion]_) and thus have a low disperser quality; carp individuals at the *bottom right* corner of the graph are likely to ingest and transport seeds intact to new locations (i.e. high *P*_[ingestion]_, low *P*_[digestion]_), having a high disperser quality. **b** The dispersal probabilities for each carp, calculated as *P*_[dispersal]_ = *P*_[ingestion]_ × (1 − *P*_[digestion]_), showing the continuous variation in disperser quality among the 12 individuals. **c** Individual differences in the shape of the dispersal kernels for *S. emersum* (*left*) and *S. sagittifolia* (*right*) seeds among 12 carp. The dispersal kernels were modelled based on seed excretion rates over a period of 24 h (faeces were collected every 2 h), assuming non-stop, linear swimming at a constant optimum speed of 1.25 body lengths s^−1^ (or 1.125 km h^−1^). To correct for potential biases towards the right limit of the observation intervals, excretion events were assigned to the mid-value of each time interval

The variation in seed ingestion and seed survival during gut passage translates into remarkably large individual differences in disperser quality among the 12 carp individuals. These differences are summarized in Fig. 4. Figure 4a graphically shows the differences in dispersal capacity among the carp individuals. At one end of the spectrum we find individuals that have a high tendency to ingest seeds (i.e. high *P*_[ingestion]_) and a low tendency to digest these seeds (low *P*_[digestion]_). These individuals are thus likely to eat seeds and transport these intact to new locations and can therefore be typified as having a high disperser quality. At the other end of the spectrum we find individuals that rarely eat seeds (low *P*_[ingestion]_) and digest most of the ones they ingest (high *P*_[digestion]_). These latter individuals have a low disperser quality (Fig. 4a). A similar negative correlation between ingestion and digestion was observed in waterfowl, at an inter-specific level. Figuerola et al. (2002) analysed bird droppings collected in the field in the Doñana wetlands (south–west Spain) and found that duck and coot species that tended to ingest more widgeongrass seeds (*Ruppia maritima*) also tended to produce droppings with a smaller fraction of digested seed fragments and, hence, a larger proportion of seeds that survived gut passage. Together these findings suggest that this may reflect a general phenomenon in the digestive processing of seeds by zoochoric dispersers that occurs both at an intra- and inter-specific level: the more seeds an animal ingests, the less efficient its digestion.

The probability of carp-mediated seed dispersal can also be calculated for each individual as: *P*_[dispersal]_ = *P*_[ingestion]_ × (1 − *P*_[digestion]_). This too reveals large differences in seed dispersal potential between carp individuals: There is more than a 31-fold difference in dispersal probability of *S. emersum* seeds among carp individuals (ranging from *P*_[dispersal]_ = 0.01995 for carp 3 to *P*_[dispersal]_ = 0.62106 for carp 5) and a nearly sevenfold difference in the dispersal probability of *S. sagittifolia* seeds (ranging from *P*_[dispersal]_ = 0.05297 for carp 3 to *P*_[dispersal]_ = 0.35750 for carp 5) (Fig. 4b). The differences between the two plant species are most likely related to differences in seed morphology (Pollux et al. 2006).

Figure 4c shows individual differences in the shape of the dispersal kernels. These kernels were modelled by combining information on seed retention times over a period of 24 h with information on the optimum swimming speed (*U*_opt_; defined as that at which the energy required per unit of distance travelled is minimized) of the common carp (Beamish 1978; Pollux 2011). The optimum swimming speed for the common carp in our experiment (with a body mass of ca. 300 g and body length of ca. 0.25 m) is close to 1.25 body lengths per second or 1.125 km per hour (Ohlberger et al. 2006). The kernels depicted in Fig. 4c assume non-stop, linear swimming at a constant optimum speed of 1.125 km per hour and herewith provide information about differences in the maximum potential distances over which the different carp can disperse seeds (Pollux 2011). The models reveal a more than twofold difference in maximum potential dispersal distances among carp individuals, ranging from 7.875 to 16.875 km for *S. emersum* seeds and 7.875 to 19.125 km for *S. sagittifolia* seeds over a period of 24 h (Fig. 4c). These distances are realistic, as a recent study showed that migrating common carp can swim distances of up to 21.6 km per day (Jones and Stuart 2009). Actual dispersal distances in the field may differ from those depicted in Fig. 4c, because while some carp may swim continuously in a single direction, most carp will sometimes rest or change speed or direction while swimming. Nevertheless, it is clear that some individuals have a far greater potential for long-distance dispersal than others. If one considers an individual’s maximum potential seed dispersal distance to be an important aspect of disperser quality (Albert et al. 2011; Wolf and Weissing 2012), then one can argue that this too adds to the remarkable individual differences in disperser quality among carp individuals found in this study.

### Individual differences in disperser quality under experimental versus natural conditions

This study demonstrates the existence of large, consistent differences in disperser quality among carp individuals kept under relatively homogenous laboratory conditions (i.e. relative to field conditions). One could argue that even larger differences may be found among individuals in the wild, because these latter individuals are likely to exhibit greater genetic diversity and experience far more heterogeneous environmental conditions throughout their life. Under more natural conditions, conspecific individuals are often known to actively select different food items from their shared environment (West 1986; Werner and Sherry 1987). And although the common carp is generally known to be an omnivore, some carp individuals may still prefer to eat seeds, while others are perhaps more partial to invertebrate prey (e.g. snails, crustaceans, worms or aquatic insects) or aquatic plants. This ‘individual specialization’ (or ‘niche variation’ sensu Van Valen 1965)—in which individuals each use a different subset of the population’s resource base—has been shown to be a widespread phenomenon in many animal taxa (Bolnick et al. 2003). Thus, the individual differences in disperser quality among animal individuals in the wild is likely to be even more pronounced than inferred from the present study and may have potentially important ecological, evolutionary and conservation implications (Bolnick et al. 2011; Burton et al. 2011; Wolf and Weissing 2012), both for the animal vector as well as the dispersed plant species.

## Electronic supplementary material

Below is the link to the electronic supplementary material.

Supplementary material 1 (PDF 2012 kb)
