# Supplementary material for: Consistent individual differences in seed disperser quality in a seed-eating fish
Source: Oecologia. 2016 Oct 4;183(1):81–91. doi: 10.1007/s00442-016-3749-4 (PMC5239806; doi:10.1007/s00442-016-3749-4)
Supplement: Supplementary file 1 — Supplementary material 1 (PDF 2012 kb) [file 442_2016_3749_MOESM1_ESM.pdf]

Electronic Supplementary Material

Consistent individual differences in seed disperser quality in a seed-eating fish

Bart J.A. Pollux[1]

[1]*Experimental Zoology Group, Department of Animal Sciences, Wageningen University, De Elst 1, NL-6708 WD Wageningen, the Netherlands*

Running headline: Individual differences in disperser quality

Corresponding author: Dr. B.J.A. Pollux, Experimental Zoology Group, Department of Animal Sciences, Wageningen University, De Elst 1, NL-6708 WD Wageningen, the Netherlands. E-mail: bart.pollux@wur.nl; b.pollux@gmail.com. Website: http://www.bartpollux.nl

Online Resource 1 A brief review of intra-specific variation in seed recovery percentages after gut passage inferred from seed feeding experiments with fishes, waterfowl and mammals. Given are the mean ± SE or range (between brackets) of seed recovery percentages based on *N* number of animals used in the studies. Note that none of these studies repeated the experiments and, hence, it remains unclear whether the observed intra-specific variation was consistent (i.e. repeatable over time)

		Seed recovery (%)		
Animal species	Plant species	*N*	mean±SE (Range)	Reference
**Fishes**				
Brycon guatemalensis (Machaca)	*Ficus glabrata*	6	(68 – 99)†	Horn (1997)
Cyprinus carpio (Carp)	*Potamogeton natans*	4	(3 – 25)†	Smits et al. (1989)
	Potamogeton obtusifolius	4	(0 – 8)†	Smits et al. (1989)
	Potamogeton pectinatus	4	(0 – 15)†	Smits et al. (1989)
	Sparganium emersum (small seeds)	12	23.6 ± 7.7	Pollux et al. (2007)
	Sparganium emersum (medium seeds)	12	42.2 ± 9.1	Pollux et al. (2007)
	Sparganium emersum (large seeds)	12	71.0 ± 7.9	Pollux et al. (2007)
**Waterfowl**				
Anas acuta (Pintail)	*Ruppia maritima*	4	16 ± 15	Charalambidou et al. (2003)
Anas clypeata (Shoveler)	*Ruppia maritima*	4	22 ± 17	Charalambidou et al. (2003)
	Potamogeton pectinatus	2	(21 – 37)	Santamaría et al. (2002)
Anas crecca (Eurasian teal)	*Ruppia maritima*	4	26 ± 18	Charalambidou et al. (2003)
	Scirpus litoralis	3	69.33±29.82	Figuerola et al. (2010)
	Scirpus maritimus	3	68.33±20.96	Figuerola et al. (2010)
Anas penelope (Wigeon)	*Ruppia maritima*	5	38 ± 18	Charalambidou et al. (2003)
	Potamogeton pectinatus	3	(1 – 29)	Santamaría et al. (2002)
Anas platyrhynchos (Mallard)	*Ruppia maritima*	3	32 ± 22	Charalambidou et al. (2003)
	Berula erecta	4	41 ± 29	Soons et al. (2008)
	Carex pseudocyperus	4	5.0 ± 5.4	Soons et al. (2008)
	Chenopodium album	10	9.8 ± 2.4	Wongsriphuek et al. (2008)
	Comarum palustris	4	25 ± 32	Soons et al. (2008)
	Digitaria ischaemum	10	1.9 ± 0.6	Wongsriphuek et al. (2008)
	Echinochloa colonum	10	8.5 ± 1.7	Wongsriphuek et al. (2008)

	Echinochloa crusgalli	10	10.7 ± 3.7	Wongsriphuek et al. (2008)
	Eleocharis palustris	10	39.9 ± 4.7	Wongsriphuek et al. (2008)
	Epilobium hirsutum	4	16 ± 12	Soons et al. (2008)
	Epilobium palustre	4	26 ± 25	Soons et al. (2008)
	Eupatorium cannabinum	4	9.3 ± 11	Soons et al. (2008)
	Filipendula ulmaria	4	11 ± 1.8	Soons et al. (2008)
	Hypericum tetrapterum	4	41 ± 25	Soons et al. (2008)
	Iris pseudacorus	4	32 ± 29	Soons et al. (2008)
	Lycopus europaeus	4	53 ± 34	Soons et al. (2008)
	Lysimachia vulgaris	4	0.33 ± 0.28	Soons et al. (2008)
	Mentha aquatica	4	54 ± 17	Soons et al. (2008)
	Panicum dichotomiflorum	10	12.8 ± 3.7	Wongsriphuek et al. (2008)
	Peucedanum palustre	4	4.3 ± 4.6	Soons et al. (2008)
	Phragmites australis	4	0.25 ± 0.50	Soons et al. (2008)
	Polygonum lapathifolium	10	16.0 ± 3.6	Wongsriphuek et al. (2008)
	Polygonum pensylvanicum	10	21.3 ± 2.7	Wongsriphuek et al. (2008)
	Potamogeton pectinatus	4	2.8 ± 3.0	Soons et al. (2008)
	Rumex crispus	10	18.6 ± 3.2	Wongsriphuek et al. (2008)
	Sagittaria sagittifolia	4	1.5 ± 1.7	Soons et al. (2008)
	Schoenoplectus maritimus	10	51.1 ± 4.7	Wongsriphuek et al. (2008)
	Silene flos-cuculi	4	34 ± 17	Soons et al. (2008)
	Sparganium erectum	4	13 ± 4.3	Soons et al. (2008)
Fulica atra (Common coot)	*Scirpus litoralis*	5	83.80 ± 23.10	Figuerola et al. (2010)
	Scirpus maritimus	5	32.20 ± 16.24	Figuerola et al. (2010)
Marmaronetta angustirostris	*Scirpus litoralis*	5	73.60 ± 23.10	Figuerola et al. (2010)
(Marbled teal*)*	*Scirpus maritimus*	5	60.00 ± 16.24	Figuerola et al. (2010)
Netta rufina (Red-crested pochard)	*Scirpus litoralis*	3	63.00 ± 29.82	Figuerola et al. (2010)
	Scirpus maritimus	3	43.67 ± 20.96	Figuerola et al. (2010)
**Mammals**				
Bos Taurus (Cow)	*Acacia dudgeoni*	16	48 ± 10†	Razanamandranto et al. (2004)
	Acacia seyal	16	46 ± 22†	Razanamandranto et al. (2004)

	Burkea Africana	16	87 ± 13†	Razanamandranto et al. (2004)
	Enterolobium cyclocarpum	2	(78 - 86)	Janzen (1982)
	Prosopis Africana	16	90 ± 8†	Razanamandranto et al. (2004)
Equus ferus caballus (Horse)	*Enterolobium cyclocarpum*	3	(17 - 56)	Janzen (1982)
Oryctolagus cuniculus (Rabbit)	*Lithrea caustica* (single gut passage)	8	(76 - 90)	Castro et al. (2008)
	Lithrea caustica (double gut passage)	8	(40-62)	Castro et al. (2008)
Ovis aries (Sheep)	*Acacia dudgeoni*	16	2 ± 2†	Razanamandranto et al. (2004)
	Acacia seyal	16	8 ± 9†	Razanamandranto et al. (2004)
	Burkea Africana	16	5 ± 5†	Razanamandranto et al. (2004)
	Prosopis Africana	16	78 ± 20†	Razanamandranto et al. (2004)
	Trifolium campestre	10	(42-91)	Russi et al. (1992)
	Trifolium tomentosum	10	(23-53)	Russi et al. (1992)
	Trofolium stellatum	10	(13-33)	Russi et al. (1992)

† values were estimated from graphs.

Online Resource 2 Photos of the experimental procedure: **(a)** A food pellet containing five *Sparganium emersum* (top) and five *Sagittaria sagittifolia* (bottom) seeds. The pellet has a diameter of approximately 10 mm. **(b)** A carp individual that is about to voluntarily ingest an offered food pellet (the pellet is still visible in the carp's mouth)

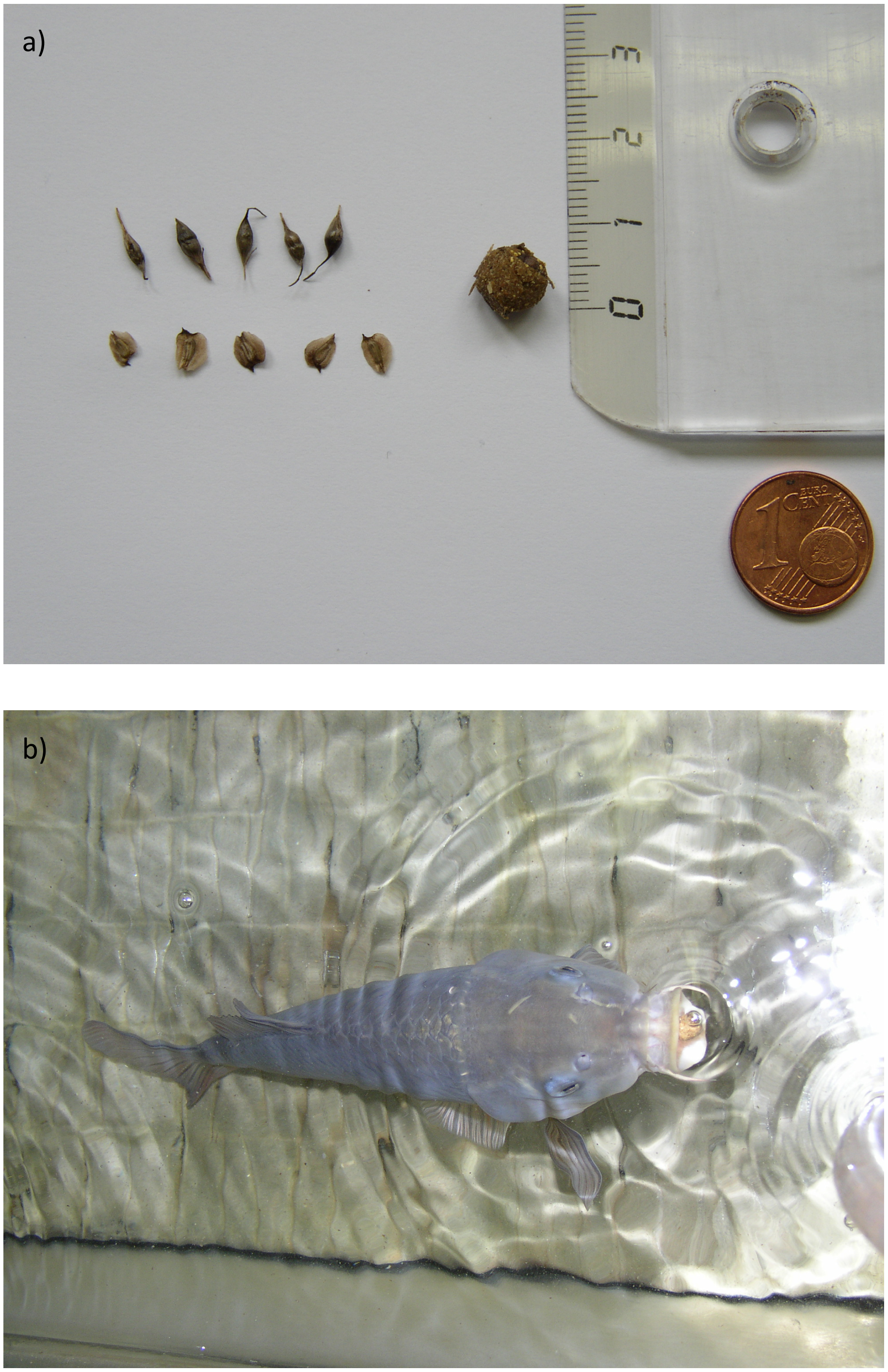

Online Resource 3 The degree of relationship of the probability of **(a)** seed ingestion, **(b)** gut survival and **(c)** germination of *Sparganium emersum* (x-axes) and *Sagittaria sagittifolia* (y-axes) seeds fed to 12 common carp (*Cyprinus carpio*). Each dot represents the mean proportion of a single carp individual and is based on *N* = 12 feeding trials. The positive correlations in graphs a and b indicate that carp individuals that, respectively, ingested and digested more *S. emersum* seeds also ingested and digested more *S. sagittifolia* seeds. Pearson correlation coefficients (*r*) and associated *P*-values are given in the text

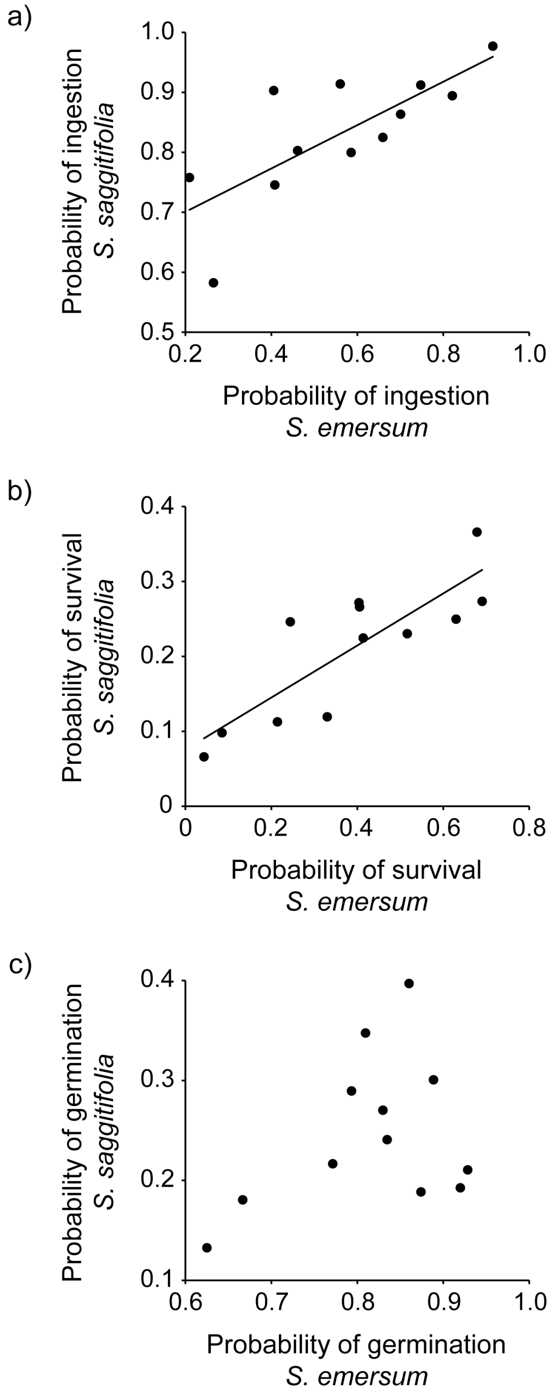

Online Resource 4 Pairwise *post hoc* comparisons of retrieval rate (Cox proportional hazards regressions using the PHREG procedure in SAS 9.2) for *Sparganium emersum* (above the diagonal) and *Sagittaria sagittifolia* (below the diagonal) among 12 carp individuals. *P*-values in bold denote a significant difference after sequential Bonferroni correction (Holm, 1979; Rice, 1989)

	1	2	3	4	5	6	7	8	9	10	11	12
1	---	0.013	0.2119	0.6122	0.0059	0.008	0.9366	0.9716	0.7138	0.7087	0.1467	0.2376
2	**0.0006**	---	0.9731	**<.0001**	0.557	0.7003	0.0512	**<.0001**	0.0012	0.001	0.8314	0.147
3	0.0012	0.0196	---	0.091	0.8548	0.8919	0.2632	0.1749	0.2577	0.1259	0.921	0.5698
4	0.0023	0.022	0.074	---	**<.0001**	**<.0001**	0.6046	0.3572	0.1753	0.961	0.0302	0.0165
5	0.0026	0.0238	0.0742	0.1413	---	0.8571	0.0301	**<.0001**	**0.0002**	**0.0003**	0.6734	0.0705
6	0.0044	0.0264	0.077	0.1607	0.2651	---	0.0365	**<.0001**	**0.0005**	**0.0005**	0.723	0.0943
7	0.0048	0.0342	0.0823	0.162	0.2808	0.5611	---	0.948	0.8267	0.6805	0.2101	0.3513
8	0.0066	0.0391	0.0993	0.1804	0.316	0.5698	0.6761	---	0.6149	0.5947	0.0904	0.106
9	0.0079	0.041	0.1009	0.1877	0.3191	0.6178	0.6972	0.8363	---	0.3839	0.1697	0.2669
10	0.0105	0.0425	0.1197	0.2124	0.3239	0.6448	0.7652	0.8678	0.9433	---	0.066	0.0869
11	0.0126	0.049	0.1248	0.2258	0.345	0.6511	0.7921	0.9185	0.9751	**<.0001**	---	0.5458
12	0.0172	0.0595	0.1303	0.2609	0.4541	0.6572	0.8357	0.9397	**<.0001**	**<.0001**	**<.0001**	---

Online Resource 5 Temporal variation in **(a)** seed ingestion, **(b)** gut survival and **(c)** germination of *Sparganium emersum* (left panels) and *Sagittaria sagittifolia* (right panels) seeds for each common carp (*Cyprinus carpio*) over the course of the twelve feeding trials (x-axes). Each line represents a single carp individual; the thick black line represents the mean of all twelve individuals. This graph shows that although there is temporal (i.e. among trial) variation (i) the differences among the individuals remain significant and repeatable (see also Figure 2) and (ii) there is no overall trend over the course of the experiment for individual carp to ingest or digest more or less seeds (e.g. due to learning behaviour, digestive plasticity or somatic growth; Charalambidou et al. 2005; Boedeltje et al. 2015)

(*figure is given below on page 9*)

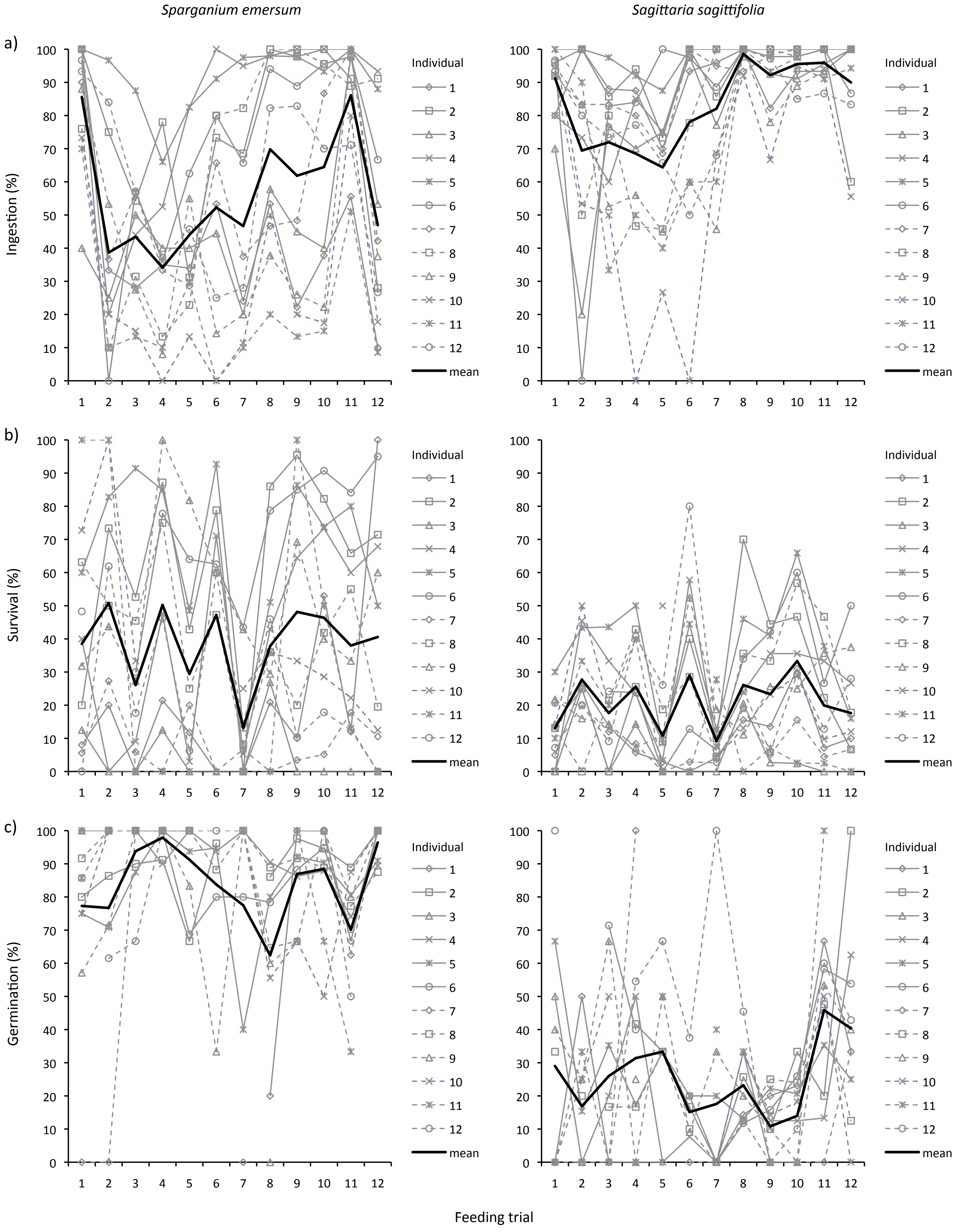

Sparganium emersum
Sagittaria sagittifolia
a)
b)
c)
Ingestion (%)
Survival (%)
Germination (%)
Individual
1
2
3
4
5
6
7
8
9
10
11
12
mean
Feeding trial
